# A comparison of artificial intelligence–enhanced electrocardiography approaches for the prediction of time to mortality using electrocardiogram images

**DOI:** 10.1093/ehjdh/ztae090

**Published:** 2024-11-18

**Authors:** Arunashis Sau, Boroumand Zeidaabadi, Konstantinos Patlatzoglou, Libor Pastika, Antônio H Ribeiro, Ester Sabino, Nicholas S Peters, Antonio Luiz P Ribeiro, Daniel B Kramer, Jonathan W Waks, Fu Siong Ng

**Affiliations:** National Heart and Lung Institute, Imperial College London, London, UK; Department of Cardiology, Imperial College Healthcare NHS Trust, London, UK; National Heart and Lung Institute, Imperial College London, London, UK; National Heart and Lung Institute, Imperial College London, London, UK; National Heart and Lung Institute, Imperial College London, London, UK; Department of Information Technology, Uppsala University, Uppsala, Sweden; Department of Infectious Diseases, School of Medicine and Institute of Tropical Medicine, University of São Paulo, São Paulo, Brazil; National Heart and Lung Institute, Imperial College London, London, UK; Department of Cardiology, Imperial College Healthcare NHS Trust, London, UK; Department of Internal Medicine, Faculdade de Medicina, and Telehealth Centre and Cardiology Service, Hospital das Clínicas, Universidade Federal de Minas Gerais, Belo Horizonte, Brazil; National Heart and Lung Institute, Imperial College London, London, UK; Richard A. and Susan F. Smith Centre for Outcomes Research in Cardiology, Beth Israel Deaconess Medical Centre, Harvard Medical School, Boston, MA, USA; Harvard-Thorndike Electrophysiology Institute, Beth Israel Deaconess Medical Centre, Harvard Medical School, Boston, MA, USA; National Heart and Lung Institute, Imperial College London, London, UK; Department of Cardiology, Imperial College Healthcare NHS Trust, London, UK; Department of Cardiology, Chelsea and Westminster Hospital NHS Foundation Trust, London, UK

**Keywords:** artificial intelligence, ECG, mortality, risk prediction

## Abstract

**Aims:**

Most artificial intelligence-enhanced electrocardiogram (AI-ECG) models used to predict adverse events including death require that the ECGs be stored digitally. However, the majority of clinical facilities worldwide store ECGs as images.

**Methods and results:**

A total of 1 163 401 ECGs (189 539 patients) from a secondary care data set were available as both natively digital traces and PDF images. A digitization pipeline extracted signals from PDFs. Separate 1D convolutional neural network (CNN) models were trained on natively digital or digitized ECGs, with a discrete-time survival loss function to predict *time to mortality*. A 2D CNN model was trained on 310 × 868 px ECG images. External validation was performed in 958 954 ECGs (645 373 patients) from a Brazilian primary care cohort and 1022 ECGs (1022 patients) from a Chagas disease cohort. The image 2D CNN model and digitized 1D CNN model performed comparably to natively digital 1D CNN model in internal [C-index 0.780 (0.779–0.781), 0.772 (0.771–0.774), and 0.775 (0.774–0.776), respectively] and external validation. Models trained on natively digital 1D ECGs had comparable performance when applied to digitized 1D ECGs [C-index 0.773 (0.771–0.774)].

**Conclusion:**

Both the image 2D CNN and digitized 1D CNN enable mortality prediction from ECG images, with comparable performance to natively digital 1D CNN. Models trained on natively digital 1D ECGs can also be applied to digitized 1D ECGs, without any significant loss in performance. This work allows AI-ECG mortality prediction to be applied in diverse global settings lacking digital ECG infrastructure.

## Graphical Abstract

**Graphical Abstract:**
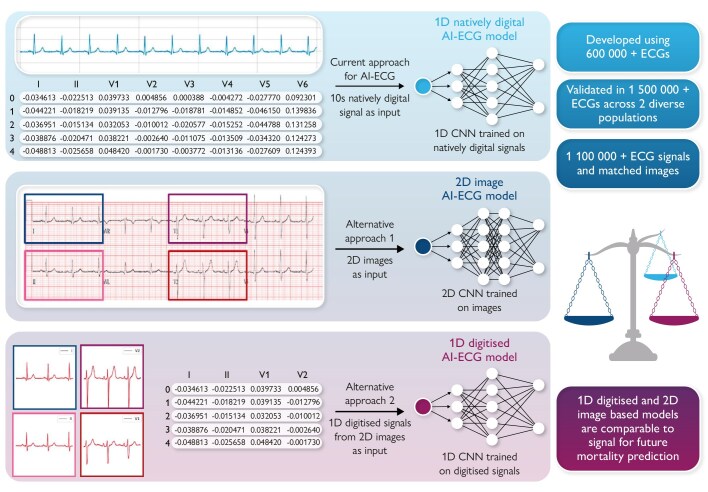

## Introduction

Electrocardiograms (ECGs) are a simple and inexpensive tool used for cardiovascular screening and diagnosis, with over 300 million ECGs performed annually.^1^ Since the emergence of convolutional neural networks (CNNs),^2^ artificial intelligence-enhanced ECG (AI-ECG) models have been shown to surpass human performance. Artificial intelligence–enhanced electrocardiogram excels in diagnosis, outperforming expert clinicians in detecting asymptomatic left ventricular dysfunction^3^ and more.^4,5^ A fundamental goal in clinical medicine is reducing the risk of adverse outcomes, including death. Artificial intelligence–enhanced electrocardiogram models can accurately identify subjects at high risk of death, for enhanced care based on clinical context.^6,7^ Predicting personalized mortality risk trajectories enables early detection of high-risk patients, allowing clinicians to target interventions effectively.

However, most existing AI-ECG algorithms rely on natively digital ECG waveforms as inputs, in an Extensible Markup Language (XML) or similar format. Currently, many clinical settings around the world lack digital ECG infrastructure but instead store ECGs as Portable Document Format (PDF) images or as paper ECGs.^8^ Older cohort studies, providing otherwise invaluable longitudinal information, may also only maintain ECGs in paper or PDF formats. As no image-based mortality prediction AI-ECG model currently exists, many clinical settings around the world without digital ECG infrastructures are deprived of using AI-ECG models for mortality prediction. Building digital ECG infrastructures incurs significant expense^9^ and is therefore typically only available in middle to higher income countries or urban settings.^10^ This may exacerbate existing healthcare inequalities for specific patient groups, such as those of lower socioeconomic status and ethnic minorities.^11^

There are broadly two approaches for the application of deep learning to images of ECGs. Firstly, the image of the ECG can be input directly into a 2D CNN architecture.^12,13^ Second, using a digitization pipeline,^14^ the ECG image can be converted into digital signals, and input into a 1D CNN architecture, similar to a natively digital ECG. It is unknown which of these approaches is superior and if they are comparable to natively digital ECG-based approaches.

**Figure 1:**
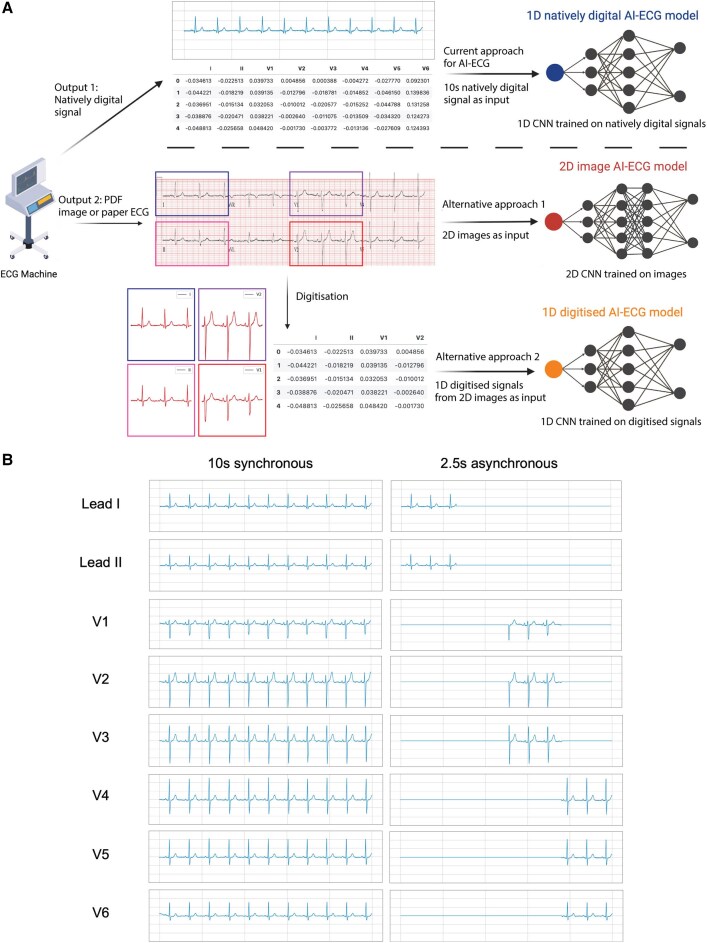
(*A*) Overview of the current standard approach for artificial intelligence–enhanced electrocardiogram using natively digital electrocardiogram signals and two novel approaches used for application of artificial intelligence–enhanced electrocardiogram to images of electrocardiograms. (*B*) Depiction of synchronous 10-s electrocardiograms (typical for natively digital electrocardiograms) and asynchronous 2.5-s electrocardiograms (typical of electrocardiogram images).

To address these questions, we developed two image-driven AI-ECG models to predict time to mortality. We compared three models: a 2D CNN using ECG images as direct input, which we will refer to as ‘image 2D CNN’; a 1D CNN using digitized ECG signals extracted from ECG images as inputs, which we will refer to as ‘digitized 1D CNN’; and the current gold standard 1D CNN using natively digital ECG signals as inputs, which will be referred to as ‘natively digital 1D CNN’ (*Figure 1A*). We performed external validation in diverse populations across different ECG image formats.

## Methods

### Ethical approvals

This study complies with all relevant ethical regulations; further details are provided in the Supplementary material online, *Methods*.

### Cohorts

We studied three cohorts. Briefly, the Beth Israel Deaconess Medical Center (BIDMC) cohort is a secondary care data set comprised of routinely collected data from Boston, USA. The São Paulo-Minas Gerais Tropical Medicine Research Center (SaMi-Trop) is a cohort of patients with chronic Chagas cardiomyopathy.^15^ The Clinical Outcomes in Digital Electrocardiography (CODE) cohort is a Brazilian database of ECGs recorded in primary care.^16^

In the BIDMC cohort, all ECGs were available as both natively digital signals of 10 s for all 12 leads and a PDF image format. The PDFs featured coloured (RGB) images with 12 leads of 2.5 s each, organized into 4 columns and 3 rows, with 3 additional 10-s leads below. The original image resolution of PDF images is 2200 × 1700 px. General Electric (Boston, USA) ECG machines were used for collection of BIDMC ECGs, and PDF images were exported using the General Electric MUSE ECG system. All cohorts had linked mortality data. Further details are provided in the Supplementary material online, *Methods*.

For model training, the BIDMC data set served as the derivation data set. The data were divided into training, validation, and testing using a 50/10/40 ratio, respectively. Electrocardiograms without corresponding 30-day life status were omitted. To ensure balanced representation, the data were split by patient ID, stratified by availability of ECGs paired with 5-year life status. To prevent data leakage, ECGs from a single patient were exclusively assigned to one of the training, validation, or test sets. The same splits were used to train all models.

### Artificial intelligence–enhanced electrocardiogram model trained using natively digital 1D electrocardiograms

Natively digital signals were pre-processed with a band-pass filter 0.5–100 Hz, a notch filter at 60 Hz, and re-sampling to 400 Hz. These parameters were based on previous studies applying AI-ECG to large data sets.^17^ Zero padding was added to make the input shape a power of 2. This resulted in 4096 samples for each lead for a 10-s recording (4000 samples + 48 zeros at the start and end), which was used as input to the neural network. As leads III, aVL, aVR, and aVF are linear combinations of leads I and II, these leads were not used for model development or evaluation. Therefore, the final input shape of a single digital ECG was 4096 × 8.

For the natively digital 1D CNN, we used a previously described CNN architecture based on residual blocks^17^ and adapted the final layer to accommodate a discrete-time survival model.^18^ 1D CNN refers to the use of 1D convolutional operations. In this study, we did not explore different 1D CNN architectures, instead choosing to focus on an established and widely used architecture.^17^ The discrete-time survival approach allows the model to account for both time to outcome (mortality) and censorship (i.e. loss to follow-up). For natively digital 1D CNN, two separate models were trained (synchronous 10 s and asynchronous 2.5 s models). Ten-second models used 10-s data simultaneously across all ECG leads, while 2.5 s models used 2.5 s of data per lead at the appropriate time points in a standard 4 × 3 ECG layout (depicted in *Figure 1B*). Zero padding was used for the sections of each lead with no signal. All 1D CNNs therefore retained the input length of 4096 per lead.

### Artificial intelligence–enhanced electrocardiogram model trained using 2D electrocardiogram images

All PDFs from the BIDMC cohort were converted to black and white images. These images were cropped to eliminate the three additional 10-s rhythm strips, resulting in a rectangular image consisting of 12 leads arranged in 4 columns and 3 rows, each containing 2.5 s of data. Rhythm strips were removed to prevent reliance on this part of the image, as these may not exist in all ECG formats. Subsequently, images were resized to 11 different resolutions from 1 × 1 to 310 × 868 px using the Python Image Library.^19^ Original ECG images were unavailable for the CODE and SaMi-Trop cohorts. Instead, digital signals represented as an array of numbers were used to generate ECG images with the ecg-plot Python library.^20^ These ecg-plot images, resized to 310 × 868 pixels, were created with and without background grids for additional validation.

For the image 2D CNN, we developed a CNN model adapted from the EfficientNet-B3 architecture, known for its efficacy in previous image AI-ECG studies.^12,13^ 2D CNN refers to the use of 2D convolutional operations. Despite the recommended input size of 300 × 300 for EfficientNet-B3,^21^ our model achieved robust performance using rectangular input dimensions, which are more suited to conventional ECG formats. The final layer used a discrete-time survival approach as discussed above. Saliency maps were created using vanilla saliency method in tf-keras-vis.^22^ Additional details on model training and hyperparameters are described in the Supplementary material online, *Methods*.

### Artificial intelligence–enhanced electrocardiogram model trained on digitized electrocardiogram signals extracted from 2D electrocardiogram images

The digitization algorithm is based on an adaptation of the method we recently described in detail.^14^ Briefly, the paper format image is initially processed to extract the ECG traces and grid features, using predefined colour/greyscale masks. Based on the detected grid, the algorithm calculates the time/amplitude resolution for scaling and identifies the per-lead regions of interest to crop the ECG traces around the 10-s/2.5-s windows of interest. The cropped images are then pre-processed for lead name removal, trace restoration, and identification, after which the ECG signal is extracted. Finally, the resulting signal is interpolated for missing values, band-pass filtered, resampled at 400 Hz, and zero padded to match the digital ECG structure (12 leads × 10 s). Extracted signals were then used to train the digitized 1D CNN as described above for the asynchronous 2.5-s model for natively digital signals.

### Assessment and comparison of model performance

We compared the performance of the natively digital 1D CNN, image 2D CNN (using the best performing resolution), and digitized 1D CNN. To determine if existing natively digital 1D CNN can be applied to digitized signals extracted from ECG images, we additionally evaluated the performance of both the 10-s synchronous and 2.5-s asynchronous natively digital 1D CNNs on digitized 1D ECGs extracted from ECG images. For the image 2D CNN, we evaluated model performance with and without the background grid to assess the ability of the model to perform accurately in an alternative image format. Model performance was assessed using concordance index (C-index). The partial likelihood ratio test was used to compare model performance.

### Scanned paper electrocardiogram images

As proof of concept, we selected 50 random ECGs from unique individuals from the BIDMC test set who either completed 5-year follow-up or died within 5 years. We used stratified sampling to retain a representative mortality rate in this small subset. The original PDF images were printed in colour and scanned in colour at 300 dots per inch to generate scanned paper ECGs. The images then followed the same pre-processing pipeline used for training: conversion to greyscale, cropping to remove rhythm strips, and rescaled to 310 × 868 pixels.

## Results

### Electrocardiogram digitization accuracy

In the BIDMC cohort, 1 163 401 ECGs were available from 189 539 subjects. The mean follow-up period was 5.46 ± 5.81 years on a per-ECG basis and 3.41 ± 4.08 years taking a random ECG per subject. A total of 34 851 (18.4%) subjects died during follow-up (see Supplementary material online, *Table S1*). Each ECG was available in both a PDF format output and a native digital (XML) format. We performed Pearson’s correlation across the corresponding natively digital 1D ECGs and digitized 1D ECGs, across all leads. The median correlation was 0.98 (0.96–1.00), and mean absolute error was 0.014 mV (0.07–0.021 mV). A median performance digitization example is shown in Supplementary material online, *Figure S1*. Supplementary material online, *Figure S2*, shows the performance distribution.

### Artificial intelligence–enhanced electrocardiogram applied to 2D images for mortality prediction—effect of image resolution

**Figure 2:**
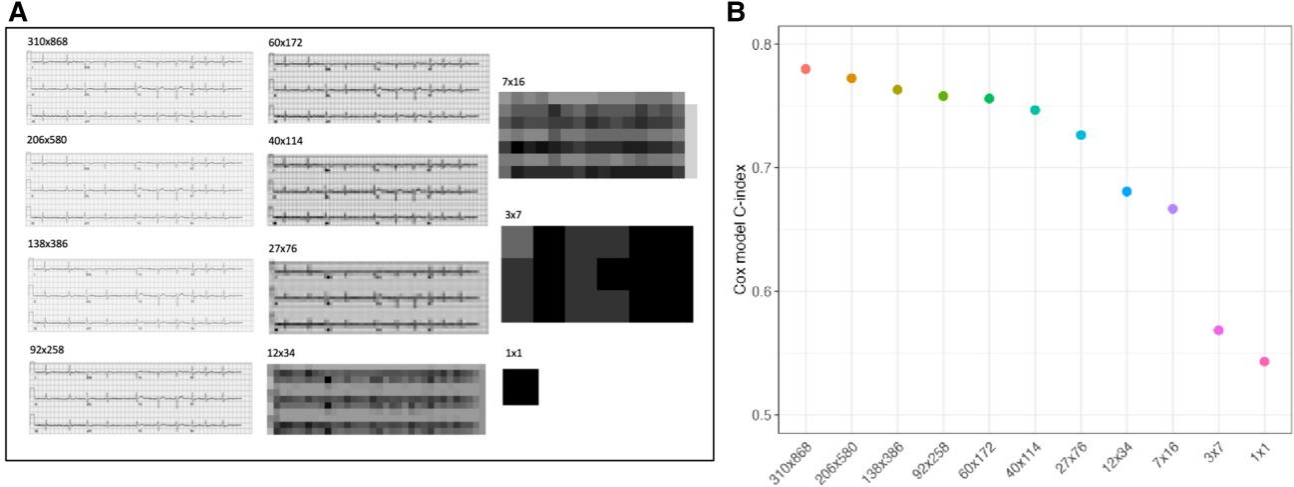
(*A*) Example images demonstrating electrocardiogram image resolutions evaluated. (*B*) Image 2D convolutional neural networks were trained at each resolution, to predict *time to mortality*. Performance as measured by the C-index was assessed for each model. Error bars indicating 95% confidence interval are too small to be seen; values are reported in Supplementary material online, *Table S1*.

**Figure 3:**
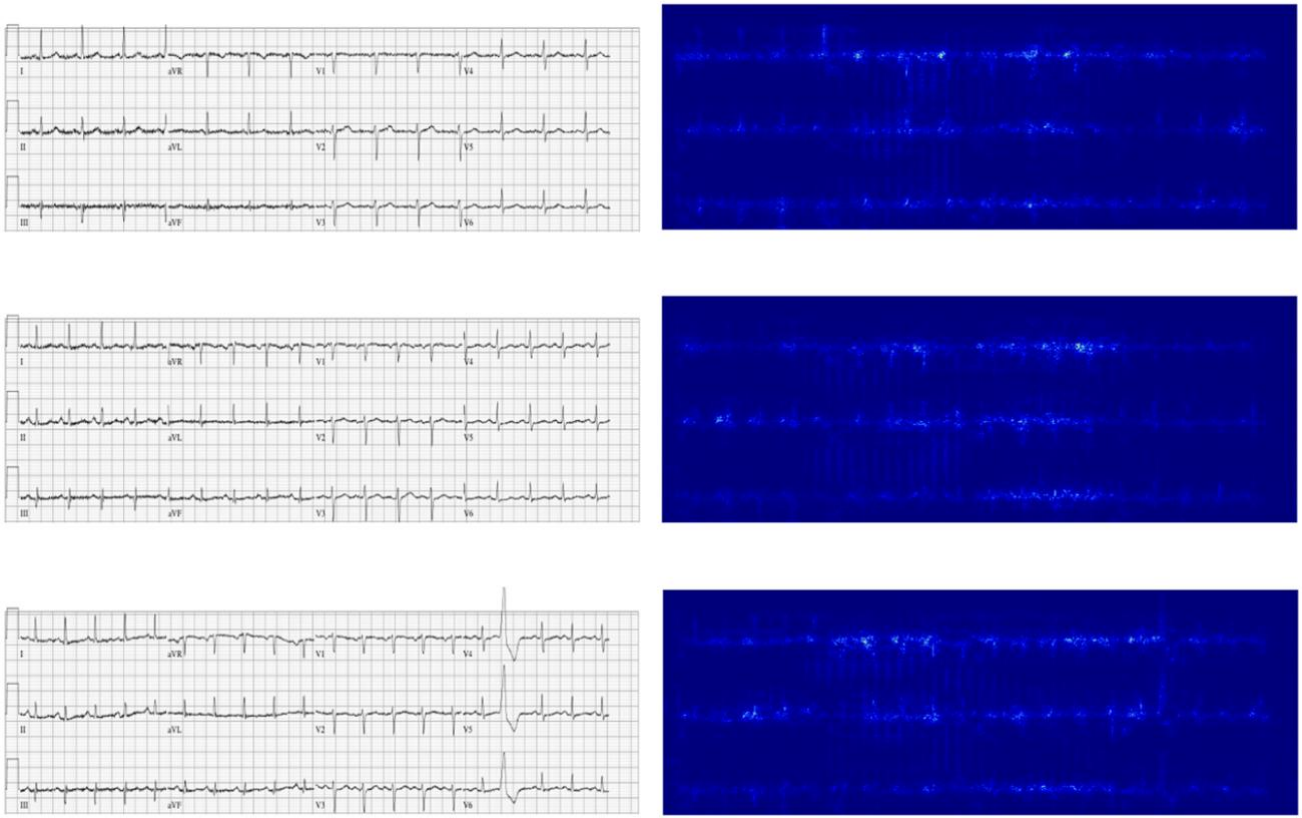
Saliency maps were plotted to demonstrate regions of the electrocardiograms most important for model predictions. Electrocardiogram signal and in particular leads aVR and V1 are highlighted in these example electrocardiograms.

We evaluated multiple image resolutions for the ECG input into the image 2D CNN, ranging from 310 × 868 to 1 × 1 px (see Supplementary material online, *Table S2*; *Figure 2A*). We found a gradual drop in performance down to a resolution of 27 × 76, with a notable drop in performance with lower resolutions (*Figure 2B*). Saliency maps of the highest resolution images are shown in *Figure 3* and demonstrate lead V1 and aVR are the most important for model predictions. Importantly, the ECG signals are highlighted demonstrating the model is not erroneously using features contained in the background grid. Saliency maps of the rest of all image resolutions is found in Supplementary material online, *Figure S3*.

### Comparison of artificial intelligence–enhanced electrocardiogram models applied to natively digital signals, 2D images, and digitized signals

**Figure 4:**
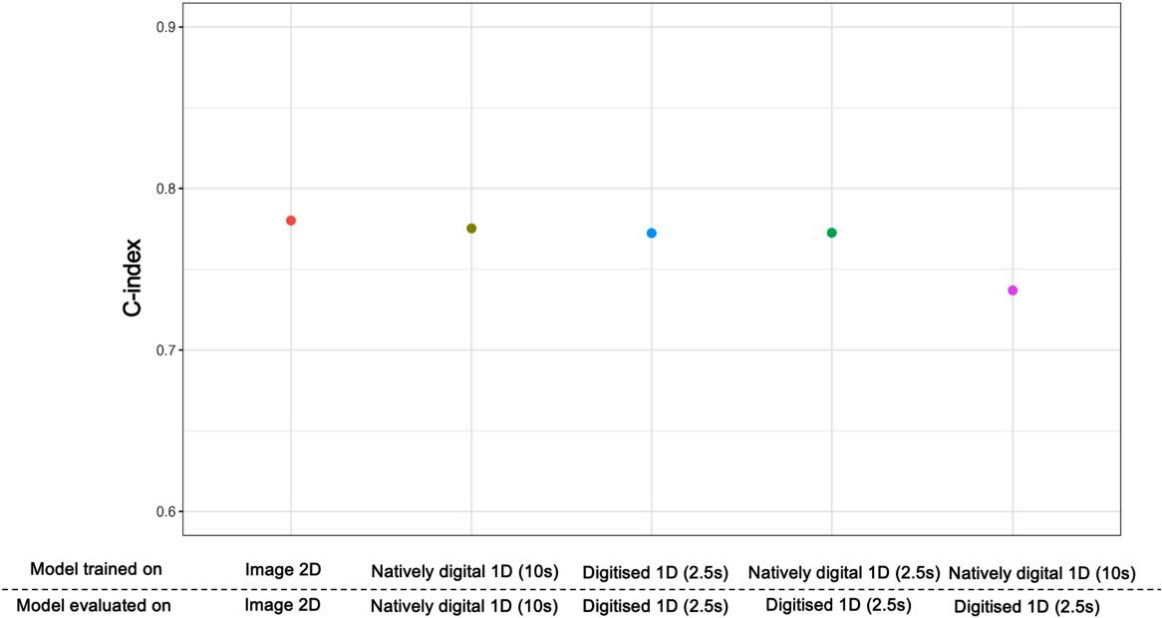
Artificial intelligence–enhanced electrocardiogram models were trained to predict mortality using natively digital electrocardiograms, 2D image electrocardiograms, and digitized electrocardiograms. Models were trained and evaluated in different five configurations as shown on the *x*-axis. The natively digital 1D convolutional neural network trained on 10-s natively digital electrocardiograms and applied on 2.5-s digitized electrocardiograms performed the worst, with the other four models having broadly similar performance. Importantly, the model trained on 2.5-s asynchronous natively digital electrocardiograms performed equivalently to the model trained on 2.5-s asynchronous digitized electrocardiograms, when both were evaluated on digitized electrocardiograms. Performance as measured by the C-index was assessed in the hold-out test set for each model. Error bars indicating 95% confidence interval are too small to be seen; values are reported in Supplementary material online, *Table S2*.

We used the best performing image 2D CNN (image resolution 310 × 868) for comparisons with other models. The image 2D CNN had the numerically highest performance but was similar to the model trained on 10-s synchronous data and the digitized 1D CNN trained on 2.5-s asynchronous data (all C-indices are between 0.77 and 0.78; Supplementary material online, *Table S3*; *Figure 4*). The natively digital 1D CNN trained on 2.5-s asynchronous data performed equally well when tested on digitized 1D ECGs with 2.5-s asynchronous data (*P* = 0.165). However, the natively digital 1D CNN trained on 10-s synchronous data and evaluated on digitized 1D ECGs with 2.5-s asynchronous data was notably inferior to the other four models [C-index 0.737 (0.736–0.738), *P* < 0.0001].

### External validation

**Figure 5:**
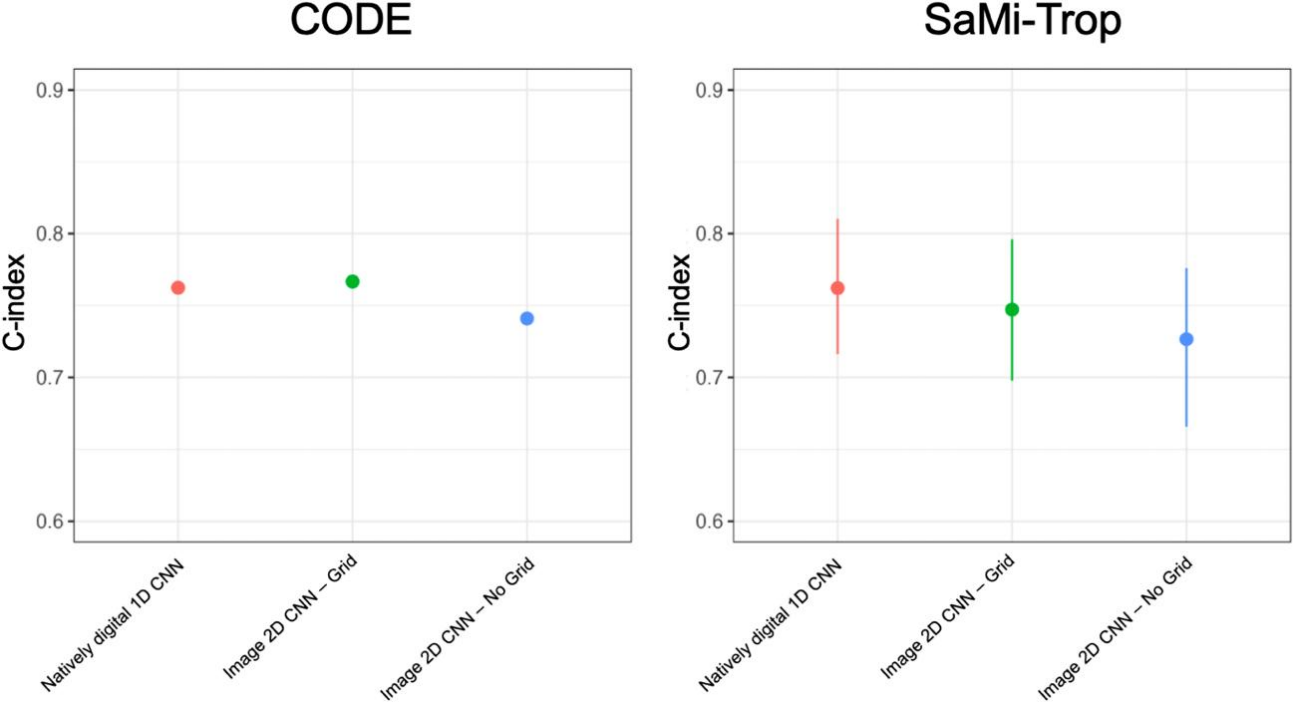
Natively digital 1D convolutional neural network and image 2D convolutional neural network models were externally validated in the CODE and SaMi-Trop cohorts. The no-grid 2D electrocardiogram image format was evaluated as an additional image format. Performance as measured by the C-index was assessed for each model. Error bars indicating 95% confidence interval are too small to be seen in CODE; values are reported in Supplementary material online, *Table S4*.

We went on to externally validate the best performing image 2D CNN (310 × 868 resolution), from the comparison of AI-ECG models above, and compared performance to natively digital 1D CNN. External validation was performed in the CODE and SaMi-Trop data sets on ECG images with and without background grid (*Figure 5*). Image examples are shown in Supplementary material online, *Figure S4*. Data set demographics for both cohorts are shown in Supplementary material online, *Table S1*.

The image 2D CNN had a slightly better performance than the natively digital 1D CNN in CODE, with no differences between these two CNNs in SaMi-Trop [CODE: natively digital 1D CNN 0.762 (0.760–0.765) vs. image 2D CNN 0.767 (0.764–0.769), *P* < 0.0001; SaMi-Trop: 0.762 (0.716–0.810) vs. 0.747 (0.698–0.796), *P* = 0.28] (see Supplementary material online, *Table S4*; *Figure 5*). We additionally compared performance of the image 2D CNN on images with no background grid (see Supplementary material online, *Table S4*; *Figure 5*) and found a minor reduction in performance (CODE, *P* = <0.0001; SaMi-Trop, *P* = 0.002).

In a subset of 50 ECGs, we printed and scanned the BIDMC test set 2D ECGs to replicate application of the model in settings without digital PDFs. In this subset, performance for mortality prediction was comparable to using natively digital 1D ECGs {image 2D CNN C-index [0.782 (0.647–0.916)] vs. natively digital 1D CNN 0.825 [0.728–0.914, *P* = 0.15]}. Example scanned images are shown in Supplementary material online, *Figure S5*.

## Discussion

In this study, we present the first comprehensive comparison of distinct approaches for AI-ECG mortality risk prediction. We compared AI-ECG applied to natively digital ECG signals with two methods for applying AI-ECG to images. One approach takes the ECG image directly and uses a 2D CNN, while a second approach digitizes the ECG image into 1D signals and then uses a 1D CNN architecture for model training and inference. We found that both approaches were comparable to natively digital ECG-based models. We additionally demonstrated that models trained on natively digital signals can be applied to digitized signals extracted from ECG images, without any significant loss in performance, but they should be trained using a 2.5-s asynchronous format. We also show that ECG image resolution can be reduced without significant losses in performance in an image 2D CNN.

### Importance of applying artificial intelligence–enhanced electrocardiogram to images of electrocardiograms

Although many AI-ECG models have recently been developed, with very high diagnostic and predictive accuracies,^3,4,7^ the overwhelming majority of models require natively digital traces as inputs and are therefore not usable in most clinical settings around the world. Building digital ECG infrastructures worldwide is not a trivial task, would be costly and time consuming, and may initially increase healthcare inequalities as digital solutions may first be implemented in more affluent regions. A temporary solution would be to adapt AI-ECG models to use ECG images as inputs. Despite the importance of this problem, there have been no studies directly comparing the two major approaches to using ECG images as inputs for AI-ECG models.

We explored the advantages and disadvantages of applying each approach to the clinically relevant and novel task of *time-to-mortality* prediction. Importantly, we have shown that both image 2D CNN and digitized 1D CNN models, based on ECG images, perform similarly to natively digital 1D CNN models, providing two alternative approaches to apply AI-ECG to images. Furthermore, AI-ECG models could be applied to decades-old cohorts with only ECG images to enable retrospective analyses with extended follow-up. This could unlock a wealth of data currently not used in AI-ECG models.

### Electrocardiogram digitization allows compatibility with models trained on digital electrocardiograms

In building on our digitization platform,^14^ we have shown the excellent accuracy of our digitization algorithm applied to ECG images in PDF format. A major advantage of the digitization approach is the potential for compatibility with the vast number of existing AI-ECG models trained on natively digital 1D ECGs. An ECG image can be put through one of these existing AI-ECG algorithms once the digital signals have been extracted. A challenge, however, is that most digital AI-ECG models are trained with 10-s data recorded simultaneously across all leads, whereas typically 2D ECG images are presented as asynchronous 2.5-s segments of data for blocks of leads, with one to three rhythm strips of 10-s duration. We found that natively digital 1D CNN models trained on digital 1D ECGs using 10 s simultaneously across all leads had reduced performance when used on digitized 1D ECGs with only asynchronous 2.5 s per lead. This is likely because the model trained on 10-s synchronous ECGs has ‘learnt’ features that relate to patterns across all leads simultaneously. When tested on asynchronous data, it lacks this context, leading to sub-optimal performance. However, when the natively digital 1D CNN model was trained in the appropriate lead format (asynchronous 2.5 s for each lead), the model performed equivalently on digitized 1D ECGs compared to the natively digital 1D CNN model trained and evaluated on natively digital 10-s 1D ECGs. This important finding suggests, going forward, natively digital 1D CNN models should also be trained using a 2.5-s asynchronous approach to facilitate translation to digitized images of 2D ECGs.

To our knowledge, no previous study has investigated this comparison over large data sets. Adedinsewo *et al.*^23^ performed an exploratory analysis in 10 digitized ECGs and found similar performance when models were applied to digitized ECGs or natively digital ECGs. However, this study was significantly limited by the very small sample size of 10 ECGs, and a model trained on 10-s simultaneous signals across all ECG leads was also not evaluated. Mishra *et al.*^24^ have reported success using 3200 digitized 1D ECGs, some of which were machine generated, on the relatively simple task of ST-elevation myocardial infarction, left and right bundle branch block, and T-wave abnormalities, but not for the more difficult task of prediction of time to mortality.

### An image-based 2D convolutional neural network approach is equivalent to 1D convolutional neural network trained on natively digital signals

In this study, we compared the digitization approach against treating the ECG data as native image input. Sangha *et al.*^12^ have described AI-ECG models using the 2D image as input.^13^ They propose the spatial information in printed images may outperform signal-based models, challenging the focus on ECG waveform signals. This notion prompts exploration of the advantages embedded in spatial information. Wu *et al*.^25^ advocate for the superiority of an ImageNet transfer-learned 2D CNN ECG model, demonstrating 98% accuracy, surpassing a 96% accuracy of the 1D CNN ECG model. Conversely, studies have also shown 1D signal inputs for time series to perform just as well as, or better than, 2D inputs.^26,27^

In our analysis, the image 2D CNN model performed marginally better than the natively digital 1D CNN in internal validation and performed similarly or slightly inferiorly on external validation, highlighting the potential challenges relating to diverse image formats, which may be improved in future through data augmentation during image model training. Importantly, despite this limitation, the image 2D CNN model performance was only slightly reduced on evaluation in external data sets and different ECG image formats when compared natively digital 1D CNN model.

### Electrocardiogram image resolution can be reduced without significant loss of performance

We evaluated the effect of image resolution on image 2D CNN performance to identify the minimum viable image resolution before any major changes in performance. One challenge of the image 2D CNN is the increased computational cost and training time when compared to training the natively digital 1D CNN. Furthermore, remote sites may have reduced computational capacity or internet speed for model inference or transferring image-based ECGs for cloud or offsite analysis. Using lower image resolutions has the potential to address these issues. We found that image resolution could be reduced to as low as 27 × 74 px with only minor changes in performance. This was a relatively surprising finding given that visually, this resolution loses many of the more subtle features that could be considered important for mortality prediction, such as QRS and T-wave morphology.^28–30^ The variation in saliency maps across resolutions, despite similar performance, suggests that the models are adapting their feature extraction strategies based on available information. At higher resolutions, the model may focus on fine-grained morphological details, while at lower resolutions, it may rely more on broader patterns or relative relationships between ECG components. This adaptability demonstrates the robustness of deep learning approaches in extracting relevant information from ECG data, even when presented in different formats or resolutions. While our current study focuses on mortality prediction, the robustness of model performance at lower resolutions raises interesting questions about the nature of the features being learned. Future work should investigate the performance of these models on more specific cardiovascular outcomes that may rely on subtle ECG morphological changes.

Our findings suggest that simpler, smaller models running on lower-end computers, along with using lower-resolution ECG images, could accelerate analysis, reduce computational costs, increase storage efficiency, and decrease training demands, thereby enhancing the accessibility of AI-ECG technologies.

Previous work using images from chest radiographs had similar conclusions, showing that lower resolutions were feasible without significant loss of model performance.^31^ Our findings on the robustness of model performance at lower resolutions suggest promising possibilities for deploying AI-ECG models in resource-constrained settings. While our study serves as a proof of concept, we recognize the need for further research to fully characterize the trade-offs between image resolution, model complexity, and performance across various clinical tasks. Future work should explore optimized architectures for low-resolution inputs and evaluate their performance in real-world, resource-limited environments.

### Clinical benefits of accurate mortality prediction

Predicting mortality risks offers a personalized and proactive approach to healthcare decision-making. The ability to understand an individual’s mortality risk trajectory facilitates targeted risk stratification, allowing clinicians to identify high-risk patients early on. Clinicians can implement timely and specific interventions, such as prescribing medications, suggesting lifestyle modifications, or recommending additional diagnostic tests. We recently described the use of a survival neural network architecture, creating a model with the ability to predict time of death without being constrained to a small number of time points.^7^ In this paper, for the first time, we have now successfully applied this methodology for mortality prediction in using both digitized 1D CNN and image 2D CNN models, allowing deployment of AI-ECG mortality prediction to all clinical settings regardless of access to digital ECG systems. An important question is when to use a digitization approach vs. an image 2D CNN approach. Our manuscript demonstrates the key advantages to each approach. The advantage of the digitization approach is that models trained on natively digital ECGs can be used and applied to the digitized data. This is a key point, given most AI-ECG models are currently trained on natively digital ECG data. However, it is important to note that the models should be trained in a 2.5-s asynchronous format as described above. In cases where ECGs are likely to be of low quality, an image 2D CNN approach may be preferred, as demonstrated by the robust performance even at reduced resolutions. Digitization is unlikely to be successful at these low resolutions.

### Limitations

There are some limitations to our findings. Although this work represents, to our knowledge, the largest validation of digitized 1D CNN and image 2D CNN models in the literature, further work in diverse image formats with varying lead layouts and for other clinical tasks is needed. Mortality prediction is most useful to clinicians when paired with predictions regarding other diseases that the patient may be at risk of. Work to extend the image approach to the other AI-ECG–predicted diagnoses^7^ is ongoing.

## Conclusions

We have developed AI-ECG models to predict mortality from the image of an ECG. We developed and validated both a digitized 1D CNN and image 2D CNN. We found both methods were comparable to natively digital ECGs, and each offers important advantages. We also show that natively digital 1D CNNs can be applied to digitized 1D ECG signals extracted from 2D ECG images. Our findings have important implications for the application of AI-ECG models to a wide range of settings.

## Supplementary Material

ztae090_Supplementary_Data

## Data Availability

SaMi-Trop cohort was made openly available (https://doi.org/10.5281/zenodo.4905618). The CODE-15% cohort was also made openly available (https://doi.org/10.5281/zenodo.4916206). Restrictions apply to additional clinical information on the CODE-15% and SaMi-Trop cohorts and to the full CODE cohort. Researchers affiliated to educational or research institutions may make requests to access the data sets. Requests should be made to the corresponding author of this paper. They will be forwarded to the relevant steering committee. The BIDMC data set is restricted due to ethical limitations.
