# Supplementary material for: A comparison of artificial intelligence–enhanced electrocardiography approaches for the prediction of time to mortality using electrocardiogram images
Source: Eur Heart J Digit Health. 2024 Nov 18;6(2):180–9. doi: 10.1093/ehjdh/ztae090 (PMC11914724; doi:10.1093/ehjdh/ztae090)
Supplement: ztae090_Supplementary_Data [file ztae090_supplementary_data.pdf]

A comparison of artificial intelligence-enhanced electrocardiography approaches for prediction of time-to-mortality using electrocardiogram images

Supplemental materials

Ethical approvals
For the Beth Israel Deaconess Medical Center (BIDMC) cohort ethics review and approval was provided by the Beth Israel Deaconess Medical Center Committee on Clinical Investigations, IRB protocol # 2023P000042.
The São Paulo-Minas Gerais Tropical Medicine Research Center (SaMi-Trop) study was approved by the Brazilian National Institutional Review Board (CONEP), No. 179.685/2012.
The Clinical Outcomes in Digital Electrocardiography (CODE) study was approved by the Research Ethics Committee of the Universidade Federal de Minas Gerais, protocol 49368496317.7.0000.5149.

ECG datasets
The datasets used in this study were selected in order to maximise the diversity of populations in which validation was conducted. As this was a retrospective study, no a priori sample size calculations were performed. Missing data was handled by complete-case analysis.

(i) The BIDMC cohort
The BIDMC cohort is a dataset comprised of routinely collected data from Beth Israel Deaconess Medical Center, Boston, USA. Subject over 16 years old with a valid ECG performed from 2014 to 2023 were included. Prior ECGs back to 2000 were included for these subjects. Mortality was determined via the Massachusetts Department of Public Health (DPH) and/or review of the BIDMC electronic medical record, while diagnostic International Classification of Diseases (ICD) codes were used to determine disease status. Subjects were censored at time of death or last in-person hospital contact.

(ii) The CODE Cohort
The CODE cohort is a database of 2,322,513 ECG records from 1,676,384 different patients of 811 counties in the state of Minas Gerais/Brazil from the Telehealth Network of Minas Gerais (TNMG). The cohort is linked to public mortality databases. Patients over 16 years old with a valid ECG performed from 2010 to 2017 were included. Clinical data, including medical diagnoses, were self-reported. In a 15% stratified sample of the original cohort (CODE-15) an ECG was labelled “normal” according to conventional clinical reporting and based on automated interval measurements (1).

(iv) The SaMi-Trop Cohort
The SaMi-Trop cohort is a prospective cohort of 1,631 patients with chronic Chagas cardiomyopathy and has been previously described in detail (2). Briefly, the inclusion criteria were: self-reported Chagas disease and aged 19 years or more. Digital ECGs were performed in 2011-2012 by TNMG. 83% of this cohort had abnormal ECGs (3).

Supplementary Methods

ECG pre-processing

PDF images

ECG images were originally 2200x1700 pixels and coloured (RGB). So first, we converted all BIDMC images to greyscale, then downscaled as the computational cost for images is very high and given the batch size of 32 and 1,163,401 ECGs, the maximum resolution that could be used to train the model was 620x868. Next, the 10s rhythm strips were removed which resulted in cropping the bottom half of rows for each resolution. The largest resolution used after pre-processing was 310x868. All ten other resolutions followed the same pre-processing steps.

Ecgplot images

As there were only natively digital signals available for CODE and SaMi-Trop datasets, signals were converted to images using ecg-plot Python library (4). The library allows the generation of images with (**Figure S4A**) and without (**Figure S4B**) background grid.

Model training

1D digitised and 1D natively digital signals models

The output of the model is a predicted probability of survival within each discrete time-interval. The model was trained to account for events occurring 10 years from the time of the ECG. The model was selected based on the lowest validation loss and was evaluated on the unseen test set. Hyperparameter optimisation was performed using the BIDMC validation set. Models were training for up to 50 epochs and the lowest validation loss of each training run used to evaluate model performance and select optimal hyperparameters. The hyperparameters tuned were the learning rate, batch size and discrete-time survival timepoints. Remaining hyperparameters were used as previously described without further tuning (5). Models were trained using a single Nvidia RTX 6000 on Imperial College London's high performance computing cluster. The Keras framework with a TensorFlow backend was used for neural network training and inference (6, 7).

2D image models

The architecture of the 2D image model was based on the EfficientNetB3 architecture (8). Similar to the 1D model, the output of the model is a predicted probability of survival within each discrete time-interval. The model was also selected based on the lowest validation loss and was evaluated on the unseen test set. Hyperparameter optimisation was performed using the BIDMC validation set. Models were training for up to 50 epochs and the lowest validation loss of each training run used to evaluate model performance and select optimal hyperparameters.

Scanned images

A random subset of 50 ECGs were selected from the BIDMC test set. We matched the 34.8% 5-year mortality in the BIDMC test set in the 50 random scanned sample. The original 2200x1700 RGB images (**Figure S5A**) were printed in colour and scanned on the 300 dots per inch setting on the HP Deskjet 2700 printer (**Figure S5B**). Scanned images followed the same pre-processing steps as stated above: conversion to greyscale, downsampled, removal of rhythm strips. As a result a greyscale 310x868 scanned image was created (**Figure S5C**).

Table S1
Data at the timepoint of a randomly selected ECG per subject is shown for the BIDMC, SaMi-Trop and CODE datasets.
Categorical variables n (%), continuous variables mean (SD)

	BIDMC	SaMi-TROP	CODE
N subjects	189539	1022	645373
Age	57.68 (18.69)	60.80 (12.89)	51.32 (17.60)
Follow up (years)	3.41 (4.08)	2.10 (0.42)	3.42 (1.70)
Sex (M)	90792 (47.9)	364 (35.6)	264128 (40.9)
Hypertension	74409 (39.3)	-	70409 (10.9)
Previous MI	11788 (6.2)	-	1730 (0.3)
Smoker	23343 (12.3)	-	15887 (2.5)
Diabetes Mellitus	33748 (17.8)	-	14182 (2.2)
Hyperlipidaemia	67087 (35.4)	-	7887 (1.2)
Mortality	34938 (18.4)	74 (7.2)	21216 (3.3)

Table S2
Summary of **image 2D CNN** performance in eleven different 2D ECG image resolutions. The concordance index (C-index) was calculated for each of the eleven **image 2D CNN** models.

Dataset	Model	C-index
BIDMC test - 1x1	Image 2D CNN - 1x1	0.543 (0.541-0.545)
BIDMC test - 3x7	Image 2D CNN - 3x7	0.569 (0.567-0.570)
BIDMC test - 7x16	Image 2D CNN - 7x16	0.667 (0.665-0.668)
BIDMC test - 12x34	Image 2D CNN - 12x34	0.680 (0.679-0.683)
BIDMC test - 27x76	Image 2D CNN - 27x76	0.726 (0.725-0.728)
BIDMC test - 40x114	Image 2D CNN - 40x114	0.747 (0.746-0.748)
BIDMC test - 60x172	Image 2D CNN - 60x172	0.756 (0.754-0.757)
BIDMC test - 92x258	Image 2D CNN - 92x258	0.758 (0.757-0.769)
BIDMC test - 138x386	Image 2D CNN - 138x386	0.763 (0.762-0.764)
BIDMC test - 206x580	Image 2D CNN - 206x580	0.773(0.771-0.774)
BIDMC test - 310x868	Image 2D CNN - 310x868	0.780 (0.779-0.781)

Table S3
Summary of **natively digital 1D CNN**, **image 2D CNN** and **digitised 1D CNN** performance in BIDMC test set. The concordance index (C-index) was calculated for natively digital 1D ECGs, digitised 1D ECGs and image 2D ECGs.

Evaluation Data format	Model	C-index
Natively digital ECGs (10s)	Natively digital 1D CNN (10s)	0.775 (0.774-0.776)
Image 2D ECGs	Image 2D CNN	0.780 (0.779-0.781)
Digitised ECGs (2.5s)	Natively digital 1D CNN (10s)	0.737 (0.736-0.738)
Digitised ECGs (2.5s)	Digitised 1D CNN (2.5s)	0.772 (0.771-0.774)
Digitised ECGs (2.5s)	Natively digital 1D CNN (2.5s)	0.773 (0.7710-0.774)

Table S4

Summary of **natively digital 1D CNN** and **image 2D CNN** performance in SaMi-Trop and CODE datasets. The concordance index (C-index) was calculated for both natively digital 1D ECGs and 2D ECGs. The **image 2D CNN** was evaluated on ecgplot images with and without background grid.

**Dataset**	**Model**	**C-index**
SaMi-Trop	Digital 1D CNN	0.762 (0.716-0.810)
SaMi-Trop - Grid	Image 2D CNN	0.747 (0.698-0.796)
SaMi-Trop - No Grid	Image 2D CNN	0.727 (0.666-0.776)
CODE	Digital 1D CNN	0.762 (0.760-0.764)
CODE - Grid	Image 2D CNN	0.767 (0.764-0.769)
CODE – No Grid	Image 2D CNN	0.741(0.738-0.743)

Figure S1
PDF ECG images were digitised. This is an example of median correlation (r = 0.98), native digital signal in black, digitised signal in red. There is almost perfect overlap of the signals.

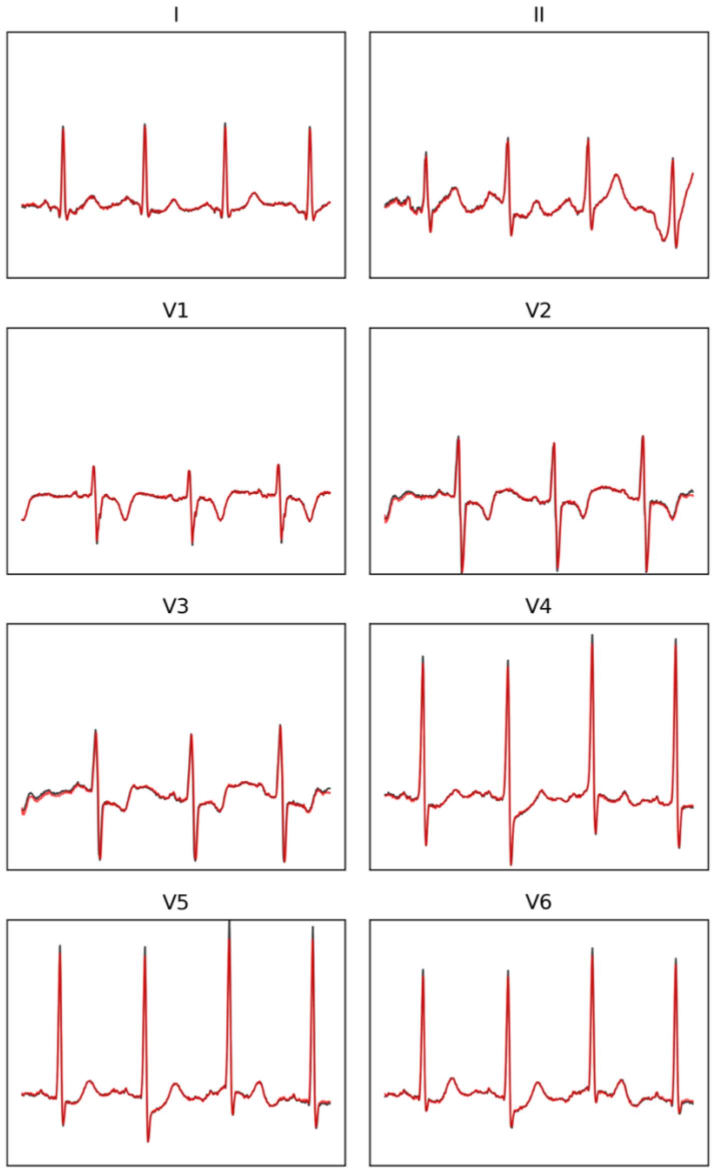

Figure S2

Pearson's correlation values across the digital and digitised ECG signals of the BIDMC cohort (median R: 0.98). Correlation is computed for all leads between the 2.5-sec windows present in both digital and paper ECG (PDF) format.

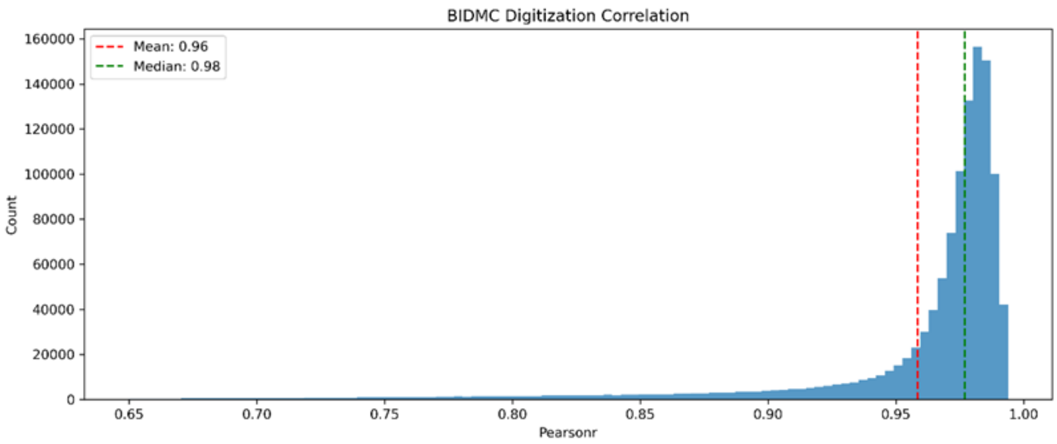

Figure S3
Saliency maps
Saliency maps of image resolutions 310x868 pixels down to 7x16 pixels of the same ECG. Highlighted areas reflect what the model focuses on in making predictions.

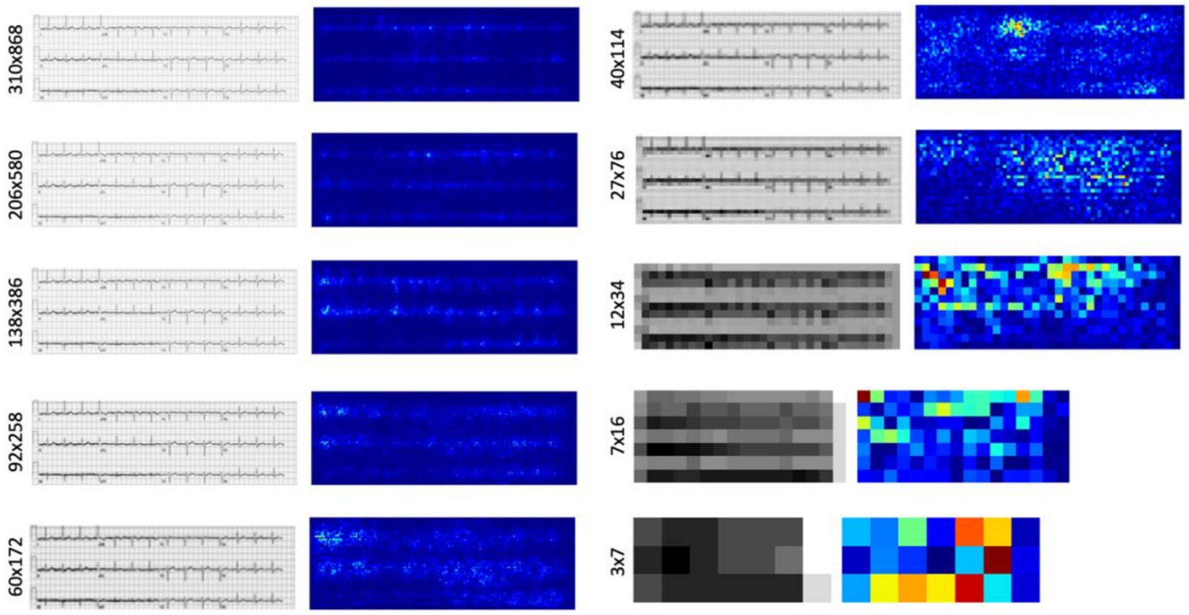

Figure S4
Example of ecgplot images created from natively digital 1D ECGs. A) SaMi-Trop ecgplot image with background. B) SaMi-Trop ecgplot image without background.

A

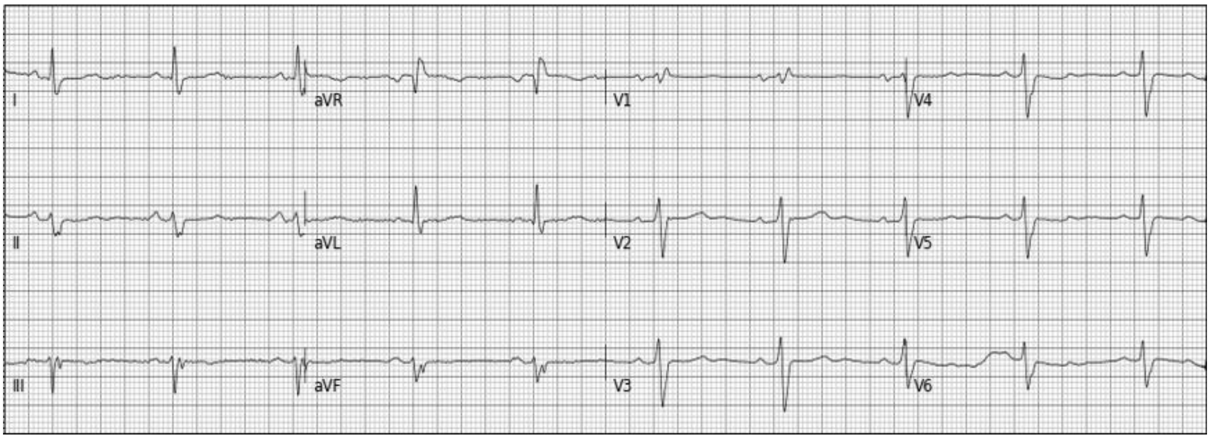

B

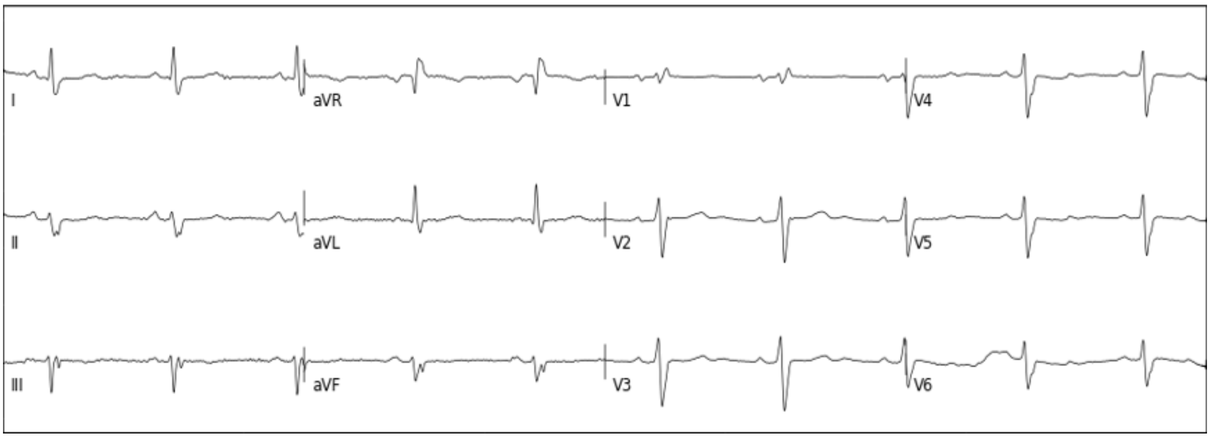

Figure S5

Scanned images examples

A random subset of 50 PDF images from the BIDMC test set were printed and scanned for external validation. A) Original PDF image. B) Scanned image in colour and 300 dots per inch. C) The final greyscale image, with rhythm strips removed and resized to 310x868 pixels, fed into the **image 2D CNN**.

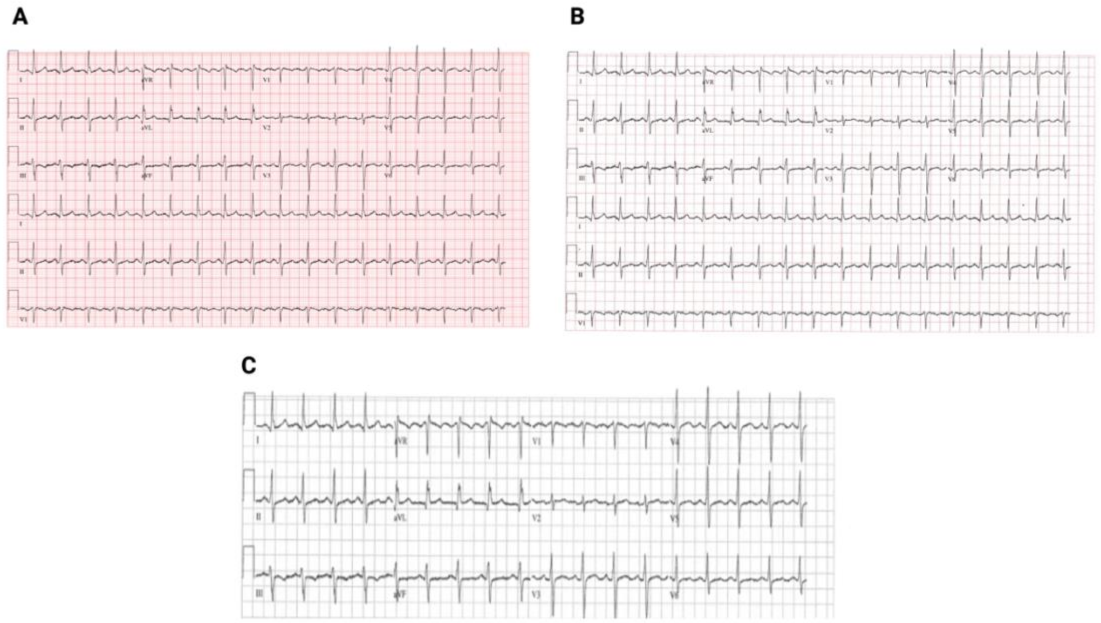

References

1. Lima EM, Ribeiro AH, Paixão GMM, Ribeiro MH, Pinto-Filho MM, Gomes PR, et al. Deep neural network-estimated electrocardiographic age as a mortality predictor. Nature Communications. 2021;12(1):5117.
2. Cardoso CS, Sabino EC, Oliveira CDL, de Oliveira LC, Ferreira AM, Cunha-Neto E, et al. Longitudinal study of patients with chronic Chagas cardiomyopathy in Brazil (SaMi-Trop project): a cohort profile. BMJ Open. 2016;6(5):e011181.
3. Cardoso CS, Ribeiro ALP, Oliveira CDL, Oliveira LC, Ferreira AM, Bierrenbach AL, et al. Beneficial effects of benznidazole in Chagas disease: NIH SaMi-Trop cohort study. PLoS Negl Trop Dis. 2018;12(11):e0006814.
4. ECG Plot Python Library [07/02/24]. Available from: https://pypi.org/project/ecg-plot/.
5. Ribeiro AH, Ribeiro MH, Paixao GMM, Oliveira DM, Gomes PR, Canazart JA, et al. Automatic diagnosis of the 12-lead ECG using a deep neural network. Nat Commun. 2020;11(1):1760.
6. Chollet F. Keras 2015 [Available from: https://keras.io.
7. Martín A, Ashish A, Paul B, Eugene B, Zhifeng C, Craig C, et al. TensorFlow: Large-Scale Machine Learning on Heterogeneous Systems. 2015.
8. Image classification via fine-tuning with EfficientNet [07/02/24]. Available from: https://keras.io/examples/vision/image_classification_efficientnet_fine_tuning/.
